# Kisspeptin-1 regulates forebrain dopaminergic neurons in the zebrafish

**DOI:** 10.1038/s41598-020-75777-0

**Published:** 2020-11-09

**Authors:** Nurul M. Abdul Satar, Satoshi Ogawa, Ishwar S. Parhar

**Affiliations:** Brain Research Institute, Jeffrey Cheah School of Medicine and Health Sciences, Monash University Malaysia, 47500 Bandar Sunway, Selangor Malaysia

**Keywords:** Neuroscience, Neural circuits, Social behaviour

## Abstract

The habenula is a phylogenetically conserved epithalamic structure, which conveys negative information via inhibition of mesolimbic dopamine neurons. We have previously shown the expression of kisspeptin (Kiss1) in the habenula and its role in the modulation of fear responses in the zebrafish. In this study, to investigate whether habenular Kiss1 regulates fear responses via dopamine neurons in the zebrafish, Kiss1 peptides were intracranially administered close to the habenula, and the expression of dopamine-related genes (*th1*, *th2* and *dat*) were examined in the brain using real-time PCR and dopamine levels using LC–MS/MS. *th1* mRNA levels and dopamine levels were significantly increased in the telencephalon 24-h and 30-min after Kiss1 administration, respectively. In fish administered with Kiss1, expression of neural activity marker gene, *npas4a* and *kiss1* gene were significantly decreased in the ventral habenula. Application of neural tracer into the median raphe, site of habenular Kiss1 neural terminal projections showed tracer-labelled projections in the medial forebrain bundle towards the telencephalon where dopamine neurons reside. These results suggest that Kiss1 negatively regulates its own neuronal activity in the ventral habenula via autocrine action. This, in turn affects neurons of the median raphe via interneurons, which project to the telencephalic dopaminergic neurons.

## Introduction

The habenula is an evolutionarily conserved epithalamic structure in vertebrates. In teleosts, the habenula is divided into the dorsal (dHb) and the ventral habenula (vHb)^1^, which send efferent outputs to different targets^2^. In the zebrafish (*Danio rerio*), the dHb can be further divided into the left and the right subnuclei that project and terminate in the ventral and the dorsal interpeduncular nuclei (IPN), respectively^1,2^. On the other hand, neurons of the vHb project to the ventro-anterior corner of the median raphe (MR), a subregion of the MR through the fasciculus retroflexus to the superior raphe^3,4^. In the zebrafish, the role of dHb-IPN pathway has been implicated in the control of aversive responses such as fear and social conflict^5–7^. However, a recent optogenetic study revealed that stimulation of vHb neurons evokes place avoidance behaviour in zebrafish^8^. We have previously demonstrated the co-expression of kisspeptin (Kiss1), a neuropeptide and its cognate G-protein coupled receptor-54 (GPR54 = Kiss1R) in the vHb of zebrafish^9^. Central administration of Kiss1 peptides significantly suppress *kiss1* gene expression, suggesting an autocrine regulation of the Kiss1 gene^10^. Further, Kiss1 has been shown to exhibit neuromodulatory role in odorant (alarm substance)-induced fear-like response^11,12^, and a more recent study showed impairment of aversion-based learning in *kiss1*-mutant fish^13^.

In mammals, the habenula conveys anti-reward and aversive information via inhibition of mesolimbic dopamine neurons^14,15^. Anatomically, neurons in the lateral habenula (LHb) of rats send efferent projections that terminate on the mesolimbic dopamine neurons. Stimulation of the LHb^16^ or exposure to aversive stimuli^17^ inhibits the activity of dopamine-containing neurons in the midbrain. In addition, optogenetic activation of the habenula-dopaminergic pathway promotes active, passive and conditioned behavioural avoidance in rodents^15^. In addition to rodents, the role of dopamine signalling in aversive response is also conserved in fish^18,19^. In the tilapia (cichlid fish) dopaminergic neurons have been suggested to mediate fear responses to novel environments^20^. Although, the above studies in vertebrates suggest a potential link between the habenula-dopamine system and aversive response, more studies are still needed to confirm these pathways of aversive response.

The present study was performed to elucidate possible interaction between the habenular Kiss1 and the dopamine system, by examining the effect of Kiss1 peptides on the expression of dopamine-related genes: tyrosine hydroxylase (*th1* and *th2*) and dopamine transporter (*dat*/*slc6a3*) using real-time PCR, and dopamine levels using LC–MS/MS in the brain of adult zebrafish. To identify the dopaminergic neuronal population responsive to Kiss1 administration, we examined the expression of neural activity-dependent marker, neuronal PAS domain-containing protein 4 a (*npas4a*) gene^21^. Finally, to examine the potential association between habenular Kiss1 neurons and dopaminergic neuronal population, we applied neural tracer in the MR, site of Kiss1 terminal projections.

## Results

### Effect of Kiss1 on dopamine-related gene expression

**Figure 1:**
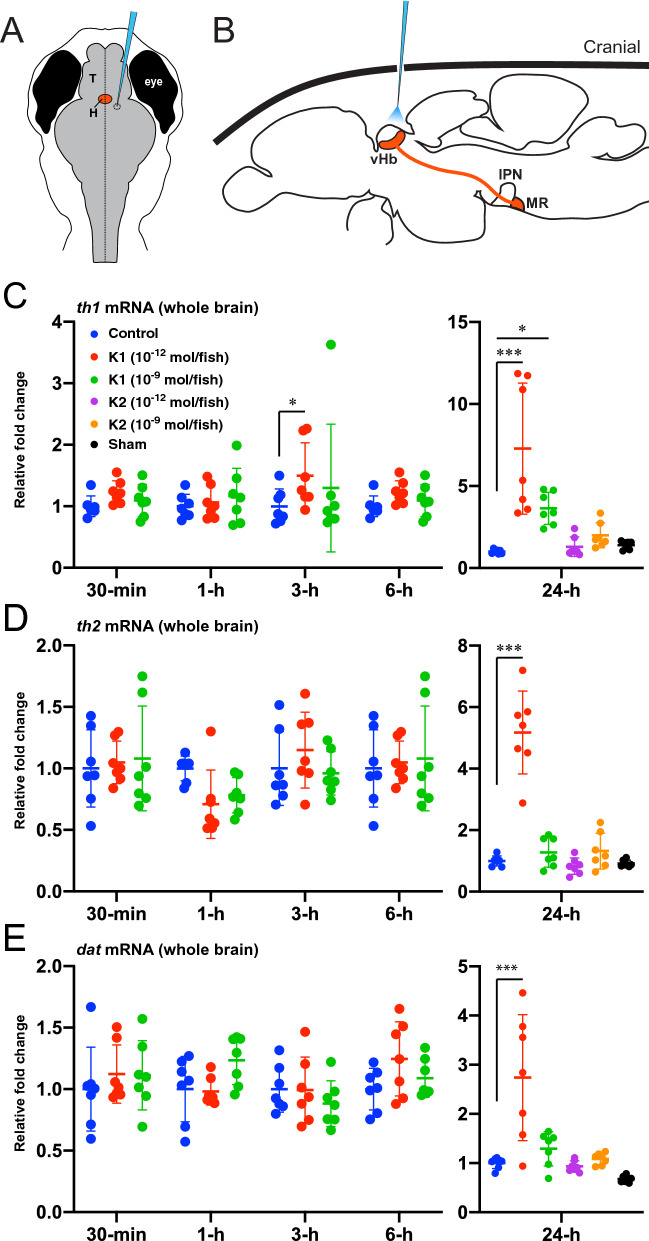
Effect of Kiss1 treatment on expression of dopamine-related genes. (**A**,**B**) Schematic drawings of horizontal (**A**) and sagittal (**B**) views of a zebrafish brain illustrating intracranial administration of Kiss1 solution. An incision is made into the skull over the anterior part of the left optic tectum using a sterilized barbed-end needle (**A**). Through the incision made, Kiss1 solution was administered nearby Kiss1 neurons in the ventral habenula (vHb) using a heat-pulled glass capillary micropipette (**B**). *T* telencephalon, *H* habenula, *IPN* interpeduncular nucleus, *MR* median raphe. Adopted from Ogawa et al. and Lim et al.^11,70^. (**C**–**E**) Expression levels of *th1* (**C**), *th2* (**D**) and *dat* (**E**) mRNAs were measured in the whole brain (n = 7) at 30-min, 1-h, 3-h, 6-h, and 24-h following the intracranial administration with either vehicle (control; distilled water) or Kiss1 (K1) or its paralogous peptide, Kiss2 (K2) at doses of 10^–12^ or 10^–9^ mol/ fish. Data are representative of mRNA expression in relative to *eef1a1l1* housekeeping gene and presented mean ± SEM as fold-change. *P < 0.05; ***P < 0.0001 versus control group using 2-way ANOVA followed by Dunnett’s multiple comparison test.

Central administration of Kiss1 peptides at dose of 10^–12^ mol/fish significantly (*P* < 0.05 or *P* < 0.0001) upregulated expression levels for *th1*, *th2* and *dat* mRNAs in the whole brain of fishes only 24-h after administration (Fig. 1C–E) but not 30-min, 1-h, 3-h or 6-h after administration as compared with those of control fishes, except for small decrease in *th1* mRNA levels in the group treated with 10^–12^ mol/fish of Kiss1 at 3-h post administration (Fig. 1C). *th1* expression was also increased at 24-h when the fish were treated with Kiss1 10^–9^ mol/fish (*P* < 0.05) (Fig. 1C). Administration with a Kiss1 paralogue, Kiss2 peptides (10^–12^ and 10^–9^ mol/fish) and sham-treatment had no effect on any of dopamine-related genes at 24-h post administration.

### Regional effect of Kiss1 on dopamine-related gene expression

**Figure 2:**
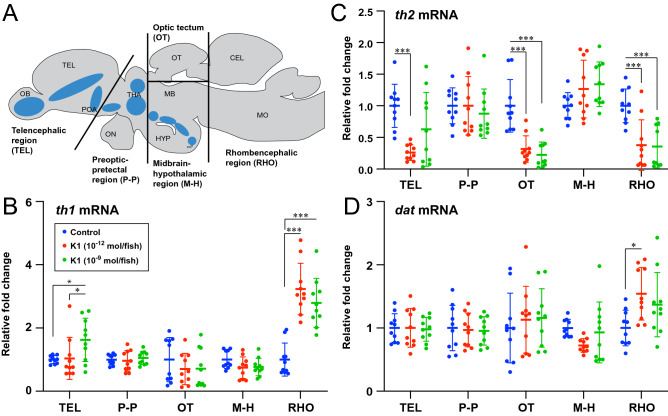
Effect of Kiss1 on the expression of dopamine-related genes in different brain regions. (**A**) Schematic sagittal view of a zebrafish brain illustrating the macro-dissected brain regions that were subjected to analyses of gene-expression levels. Blue-shaded regions indicate the distribution of dopaminergic cell populations. (**B**–**D**) Relative expression (fold-change) of *th1* (**B**), *th2* (**C**) and *dat* (**D**) mRNAs (n = 10 fish/group) in macro-dissected brain regions 24-h after central administration of either vehicle (control; distilled water) or Kiss1 (K1) with doses of 10^–12^ or 10^–9^ mol/ fish. Data presented as mean ± SD. *P < 0.05; ***P < 0.0001 using two-way ANOVA, Tukey's multiple comparisons test.

To examine the regional effect of Kiss1 administration on dopamine system, expression levels of dopamine-related genes were examined in five macro-dissected brain regions (Fig. 2A). Kiss1 administration differentially altered the expression levels of *th1* and/or *th2* mRNAs in the telencephalic, optic tecum and rhombencephalic regions but there was no change in the preoptic-pretectal and midbrain-hypothalamic regions (Fig. 2B–D).

In the telencephalic region, *th1* mRNA levels were significantly (P < 0.05) higher in fish treated with Kiss1 at the dose of 10^–9^ mol/fish as compared with control and Kiss1 at the dose of 10^–12^ mol/fish (Fig. 2B), and *th2* mRNA levels were significantly (P < 0.0001) lower in fish treated with Kiss1 at the dose of 10^–12^ mol/fish as compared to controls (Fig. 2C). In the optic tectum, there was no effect of Kiss1 administration on *th1* mRNA levels, while *th2* mRNA levels were significantly (P < 0.0001) lower in fish treated with Kiss1 at the doses of 10^–12^ and 10^–9^ mol/fish as compared to controls (Fig. 2C). In the rhombencephalic region, *th1* mRNA levels were significantly (P < 0.0001) higher in fish treated with Kiss1 at the doses of 10^–12^ and 10^–9^ mol/fish as compared to controls (Fig. 2B) and *th2* mRNA levels were significantly (P < 0.0001) lower in the fish treated with Kiss1 at the doses of 10^–12^ and 10^–9^ mol/fish as compared to controls (Fig. 2C). There was no change in *dat* mRNA levels throughout the brain regions, except in the rhombencephalic region, where *dat* mRNA levels were significantly (P < 0.05) higher in fish treated with Kiss1 at the dose of 10^–12^ mol/fish as compared to controls (Fig. 2D).

### Effect of Kiss1 on dopamine levels in macro-dissected brain

**Figure 3:**
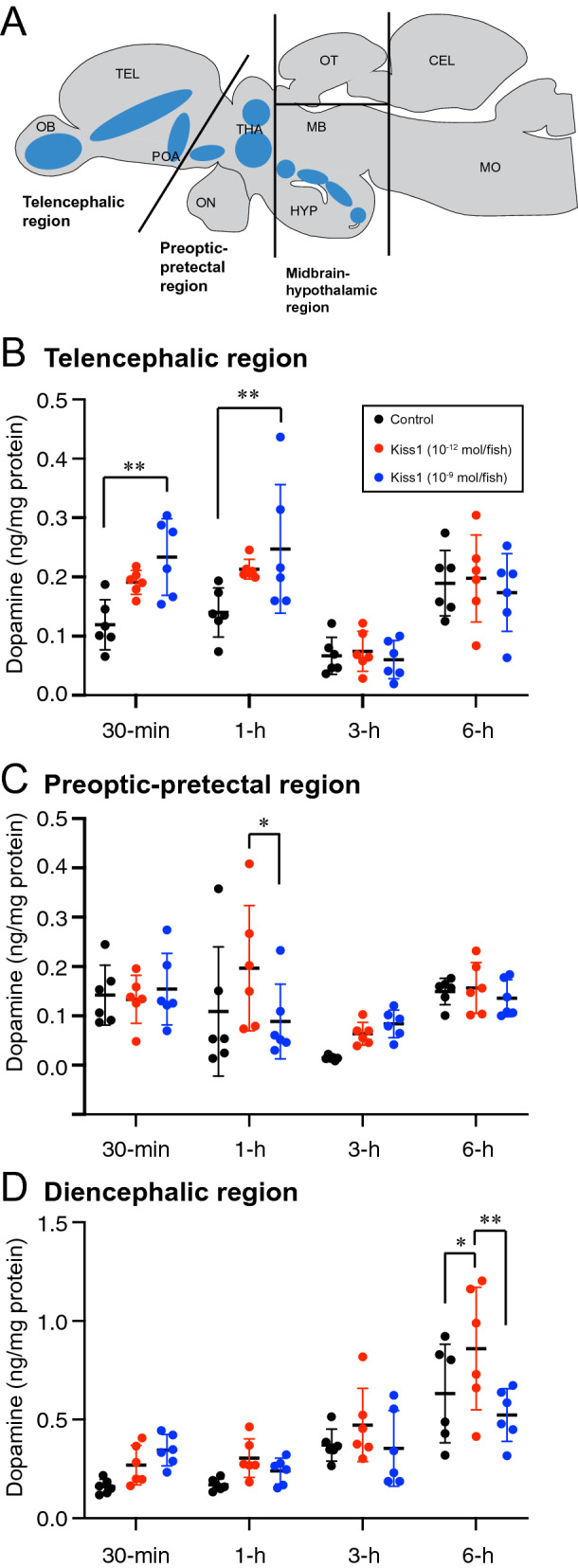
Effect of Kiss1 on dopamine levels in different brain regions. (**A**) Schematic sagittal view of a zebrafish brain illustrating the macro-dissected brain regions that were subjected to analyses of dopamine content levels and the distribution of dopaminergic cell populations (blue-shaded zones). (**B**–**D**) Dopamine content levels (ng per mg protein; n = 6 fish/group) in macro-dissected telencephalon (**B**), preoptic-pretectal (**C**) and midbrain-hypothalamic (**D**) regions, 30-min, 1-h, 3-h and 6-h after central administration of either vehicle (control; distilled water) or Kiss1 with doses of 10^–12^ or 10^–9^ mol/ fish. Data presented as mean ± SD. *P < 0.05; **P < 0.01 using 2-way ANOVA followed by Tukey’s multiple comparison test.

Dopamine (DA) levels were measured in three macro-dissected brain regions following Kiss1 administration (Fig. 3A). All groups including Kiss1-treated and controls treated with saline, absolute DA levels fluctuated time-dependently in all three brain regions (Fig. 3B–D). In the telencephalic and preoptic-pretectal regions, basal levels of DA seen at 30-min and 1-h post saline administration were decreased at 3-h (telencephalon: P < 0.0001 vs 30-min and 1-h; preoptic area, P < 0.001 vs 30-min, P < 0.05 vs 1-h) but returned to similar levels at 6-h post saline administration (telencephalon: P < 0.0001; preoptic are: P < 0.001 vs 3-h). On the other hand, in the midbrain-hypothalamic region, DA levels continued to increase from 3-h until 6-h post saline administration (P < 0.0001 vs 30-min and 1-h; P < 0.001 vs 3-h).

Kiss1 administration differentially altered DA levels in time-dependent and brain region-dependent manner (Fig. 3B–D).

*Telencephalic region*: Administration of Kiss1 at the dose of 10^–12^ mol/fish significantly increased DA levels 30-min after the administration, which further continued to increase until 1-h post administration (P = 0.0018 and P = 0.0035 for 30-min and 1-h, respectively) (Fig. 3B). There was no difference in DA levels between Kiss1 at the dose of 10^–9^ mol/fish and control at any time point.

*Preoptic-pretectal region*: There was no effect of Kiss1 administration on DA levels at 30-min, 3-h and 6-h post administration, except at 1-h post administration, where DA levels were significantly higher in fish treated with Kiss1 at the doses of 10^–12^ mol/fish as compared to fish treated with 10^–9^ mol/fish of Kiss1 (Fig. 3C).

*Midbrain-hypothalamic region*: Unlike the telencephalic or preoptic-pretectal regions, DA levels were significantly increased only 6-h after administration of Kiss1 at the dose of 10^–12^ mol/fish (Fig. 3D).

### Effect of Kiss1 administration on the habenula activity

**Figure 4:**
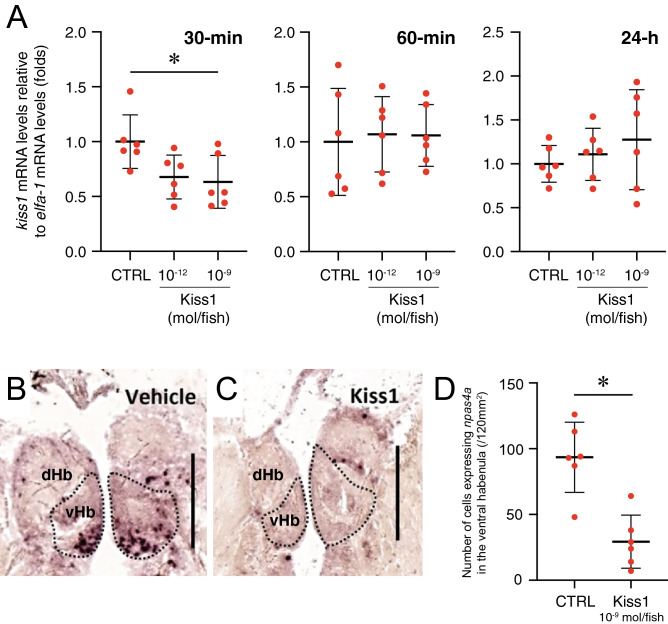
Effect of Kiss1 on the habenula activity. (**A**) Central administration of Kiss1 (10^–9^ mol/fish) significantly suppressed expression of *kiss1* mRNA levels (against the *eef1a1l1* in fold-change ± SD, n = 6 fish/ group) 30-min, but not 1-h and 24-h after administration. (**B**,**C**) Expression of *npas4a* in the vHb 30-min after the administration of vehicle (control, **B**) or Kiss1 (10^–9^ mol/fish, **C**). Scale bars 100 µm. (**D**) Number of cells expressing *npas4a* in the habenula were significantly lower as compared to control fish. *P < 0.05 vs. controls using one-way ANOVA followed by Dunnett’s multiple comparison test (*kiss1*) or unpaired *t*-test (*npas4a*).

To confirm the effect of Kiss1 administration on the habenula activity, *kiss1* gene expression was measured in the brain of fish at 30-min, 1-h and 24-h after Kiss1 administration. In fish treated with Kiss1 with dose of 10^–9^ mol/ fish, *kiss1* mRNA levels in 30-min post administration were significantly decreased as compared to vehicle controls (P = 0.0263, Fig. 4A). There was no effect of Kiss1 on *kiss1* mRNA expression in 1-h and 24-h post administration. We also examined effect of Kiss1 on neural activity in the habenula using a neural activity marker gene, *npas4a* expression. In the fish treated with Kiss1 (10^–9^ mol/ fish), number of cells expressing *npas4a* mRNA was significantly lower in the ventral habenula as compared to vehicle treated controls (P = 0.0008, Fig. 4B–D).

### Neuronal activity in dopaminergic neurons followed by Kiss1 administration

**Figure 5:**
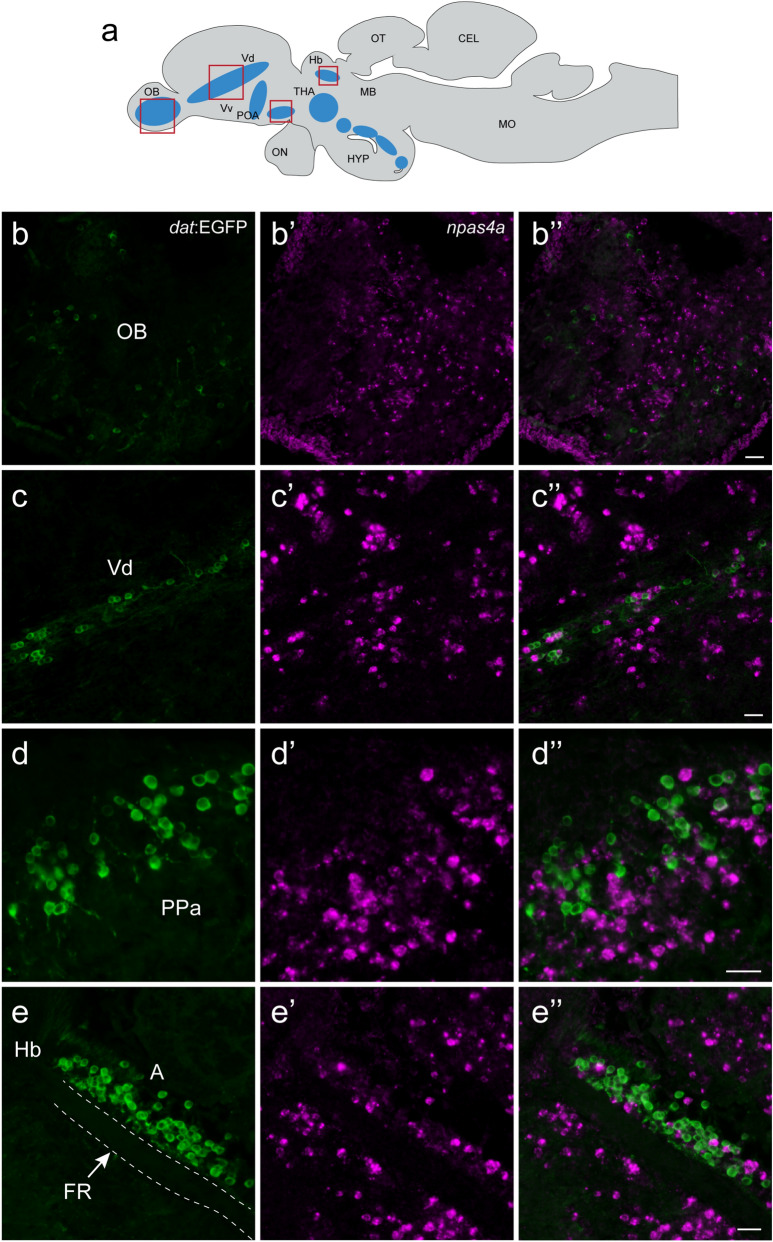
Expression of *npas4a* gene in the brain of transgenic *dat*:EGFP fish followed by 30-min post Kiss1 administration. (**a**) Schematic sagittal view of a zebrafish brain illustrating the distribution of dopaminergic cell population (blue-shaded zones) in the brain of zebrafish. Boxes with red indicate the boundaries of the photomicrographic illustrations in below (**b**–**e**). (**b**–**e**) EGFP (*green*)-labelled dopaminergic neurons in the olfactory bulb (OB, **b**); dorsal nucleus of ventral telencephalic area (Vd, **c**); anterior part of the parvocellular preoptic nucleus (PPa, **d**) and in the anterior thalamic nucleus (A, **e**) above the fasciculus retroflexus (FR). Although cells expressing *npas4a* mRNA (*magenta*) were seen in several brain regions (**b'**–**e'**), there was no expression of *npas4a* in dopaminergic cells (**b"**−**e"**). Scale bars 20 µm.

To confirm whether and which dopaminergic neuronal population (Fig. 5a) is activated by Kiss1 administration, we examined the effect of Kiss1 administration on the expression of activity-dependent neural marker, *npas4a* in dopaminergic neurons using the transgenic Tg(*dat*:EGFP) zebrafish at 30-min post administration (Fig. 5b–e). Fluorescent in situ hybridization showed expression of *npas4a* mRNA in several brain regions including the brain regions containing dopaminergic neurons (Fig. 5b'–e'). However, there was no expression of *npas4a* in EGFP-labelled dopaminergic neurons in any brain regions including the olfactory bulb (Fig. 5b"), ventral telencephalon (Fig. 5c"), preoptic region (Fig. 5d"), and the periventricular pretectal nucleus (Fig. 5e") in both control (data not shown) and Kiss1-treated fish (Fig. 5b"−e"). Similarly, no expression of *npas4a* gene was observed in other dopaminergic cell populations (data not shown).

### Neural tracing from the median raphe

**Figure 6:**
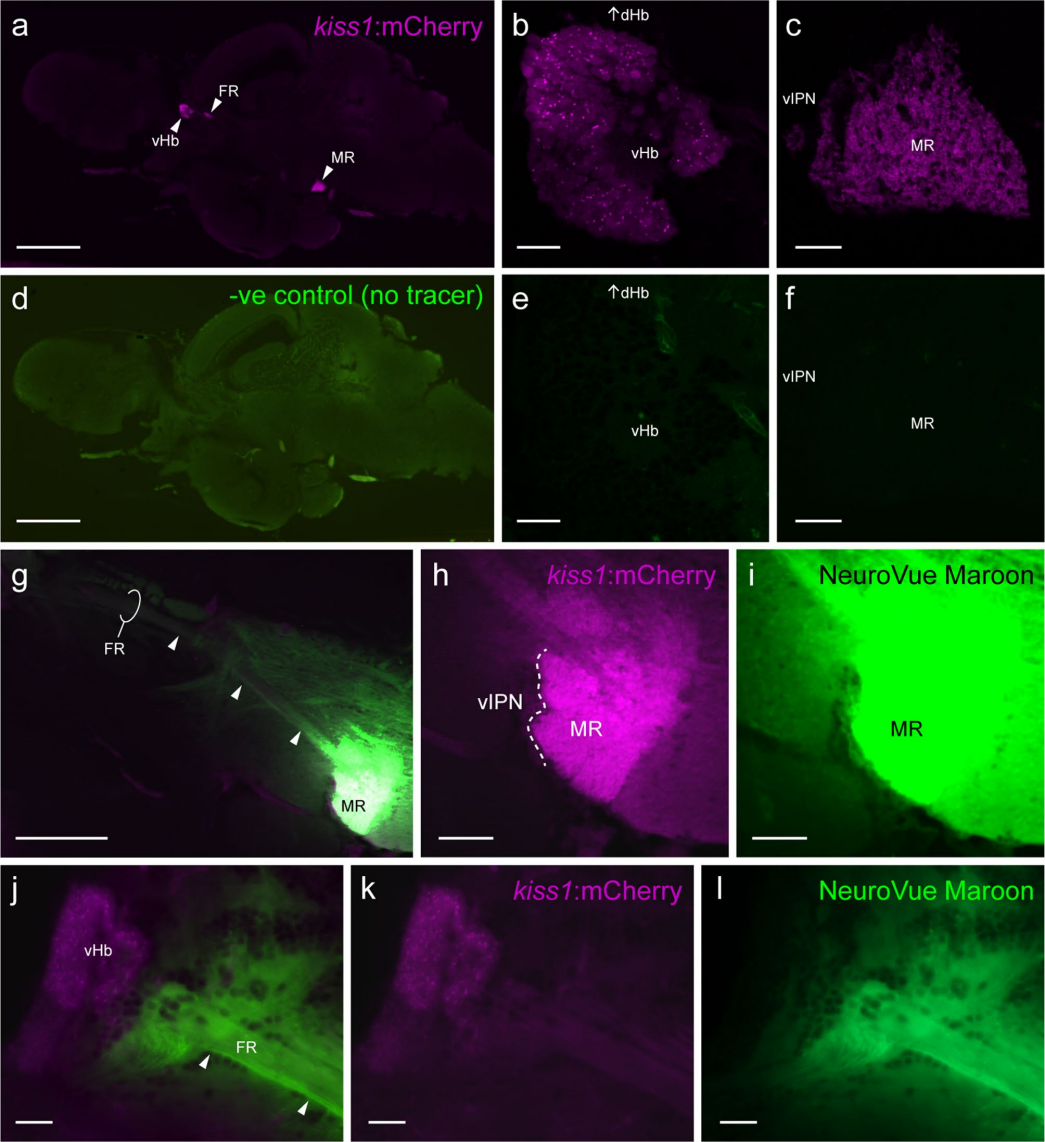
Neural tracer application into the median raphe. (**a**–**f**) Mid-sagittal visualization of mCherry (*magenta*)-labelled Kiss1 efferent projection from the ventral habenula (vHb, **b**) through the fasciculus retroflexus (FR) towards the median raphe (MR, **c**) in the brain of Tg(*kiss1*:*mCherry*) zebrafish without neural tracer implantation (**d**–**f**). (**g**–**i**) A thin-strip of the nylon membrane-coated with NeuroVue tracer (NeuroVue Maroon, *green*) was selectively inserted into the MR (**h**,**i**). (**j**–**l**) Photomicrograph of mid-sagittal visualization of tracer-labelled neural tracks (arrow heads) from the MR (**g**) towards the vHb (**j**). Scale bars (**a**,**d**) 500 μm; (**b**,**c**,**e**,**f**,**j**–**l**) 20 μm; (**g**) 200 μm; (**h**,**i**) 50 μm.

**Figure 7:**
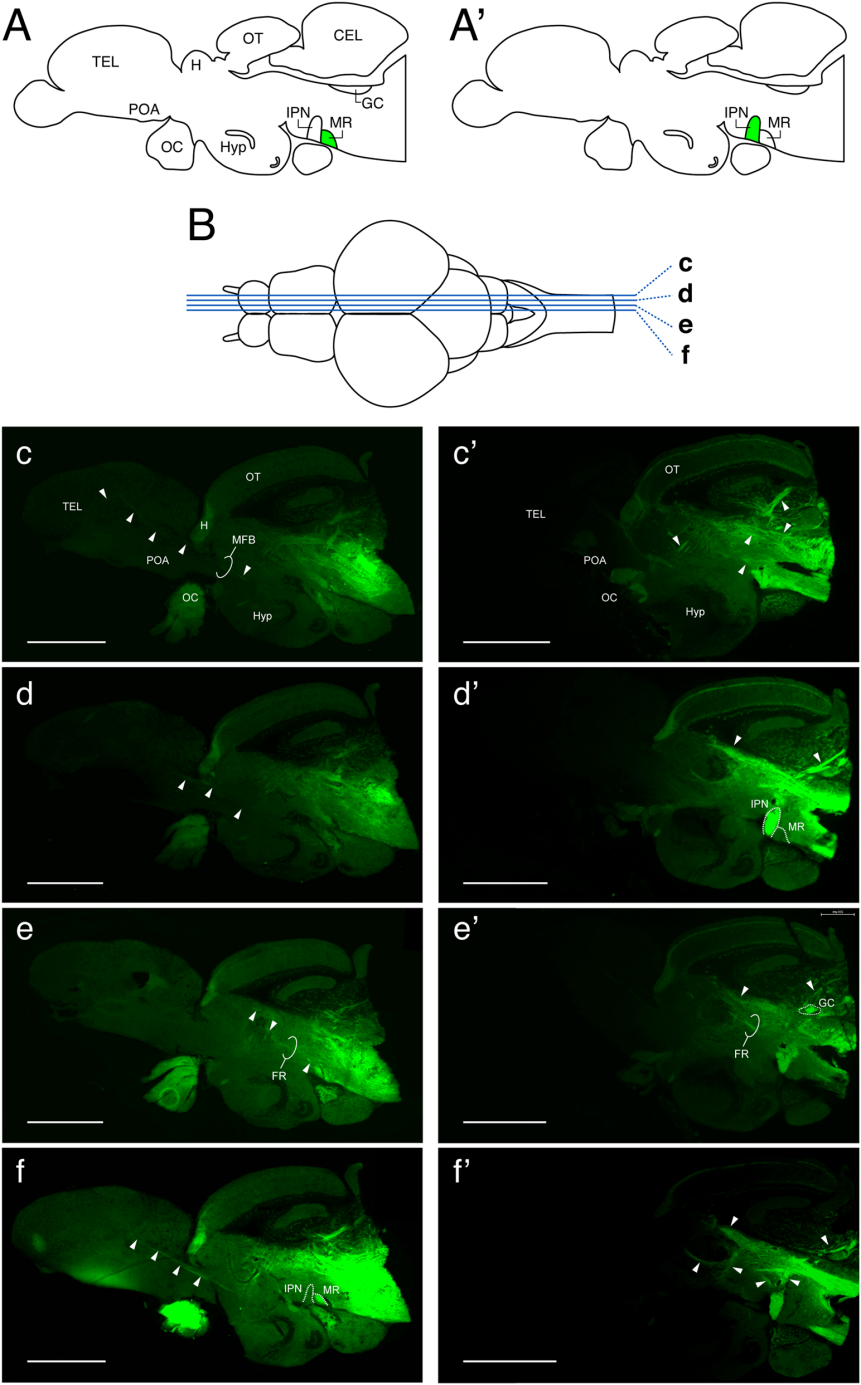
Tracer-labelled neuronal projection from the median raphe. (**A**,**A’**) Schematic sagittal view of the zebrafish brain illustrating the application sites of the neural tracer: the median raphe (MR, **A**) and interpeduncular nucleus (IPN, **A’**). (**B**) A schematic horizontal view of the zebrafish brain illustrating levels at which sagittal sections (**c**–**f** and **c’**–**f’**) were taken. (**c**–**f**) Photomicrograph of mid-sagittal visualization of tracer (NeuroVue Maroon, *green*)-labelled neural fibers/tracks (arrow heads) from the MR. Tracer-labelled fibers/tracks were seen in the mesencephalon including the fasciculus retroflexus (FR, **e**), and in the rhombencephalon (**e**–**f**). Tracer-labelled tracks were seen from the diencephalon towards the medio-dorsal region of the telencephalon which is the medial forebrain bundle (MFB, **e**,**f**). In addition, some fibers were also seen in the cerebellum (**e**,**f**). (**c’**–**f’**) In the brain where the tracer was applied to the IPN (**e’**–**f’**), tracer-labelled fibers/tracks were mainly seen in the dorsal region of the mesencephalon including the FR, and in the rhombencephalon and medulla spinal regions. In the rhombencephalon, fibers were seen in the griseum centrale (GC, **e’**). In addition, thick fibers were also seen in the cerebellum (**c’**–**f’**). Scale bars 500 μm.

To reveal the potential link between habenular Kiss1 neurons and dopaminergic neurons, a thin-strip of nylon membrane coated with neural tracer was selectively applied to the MR where habenula Kiss1 neuronal terminals are located in transgenic Tg(*kiss1*:mCherry) zebrafish (Figs. 6a–l and 7A). As negative control, we used a brain without tracer implantation (Fig. 6a–f) and a brain in which tracer was applied to the IPN region (Fig. 7A’). In the brain implanted with a tracer-coated strip in the MR (Fig. 6g–i), tracer-labelled neural tracts were seen in the fasciculus retroflexus towards the habenula (Fig. 6j–l). In addition, observation of series of the brain sections (Fig. 7B) revealed the presence of tracer-labelled nerve fibers in the medullary/brain stem regions, mesencephalic regions including the optic tectum and tegmentum, and in the optic chiasma (Fig. 7c–e). Further, distinct neural tracts were labelled in the diencephalon, which are extended towards the telencephalon. This tract is known as the medial forebrain bundle (MFB, Fig. 7c,f). In the brain implanted with a tracer into the IPN (Fig. 7A’), tracer-labelled nerve fibers were seen in the mesencephalic and rhombencephalic regions including the optic tectum, tegmentum, and cerebellum, and in the medullary regions (Fig. 7c’–f’) including the griseum centrale (GC, Fig. 7e’).

## Discussion

The role of LHb as a centre for processing negative stimuli and its-related decision-making through the modulation of midbrain dopaminergic neurons is well established in mammals^22^. Although the habenula structure and the dopamine system in the central nervous system are well conserved across the vertebrate species, the link between them remains to be identified in non-mammalian vertebrates. We have previously shown the expression of Kiss1 and Kiss1R in the vHb-MR^3,9^, and their role in fear responses^11^. In the present study, we elaborate further the possible link between habenular Kiss1 and dopaminergic neurons in the zebrafish.

Administration of Kiss1 at the dose of 10^–12^ mol/fish elevated the expression of dopamine-related genes 24-h post administration, while Kiss1 at the dose of 10^–9^ mol/fish effected only *th1* expression 3-h post administration in the whole brain. However, at the brain regional levels, expression of dopamine-related genes appeared to be differentially altered in different brain regions and different concentrations of Kiss1 solutions, suggesting that not all dopaminergic neurons are sensitive to Kiss1. *th1* gene expression was upregulated only in the telencephalon and the hindbrain, and *th2* gene expression was downregulated in the telencephalon, optic tectum and the hindbrain despite of the absence of dopaminergic neurons in some of the brain regions. On the other hand, the dopamine levels increased in the telencephalon, preoptic area and the hypothalamus after Kiss1 administration with peaks at different time points, which is inconsistent with gene expression results. At present, it is unclear how Kiss1 modulates dopamine-related genes and how these gene expression levels are associated with dopamine content levels at different times in different brain regions. Nevertheless, differential effects of Kiss1 on dopamine-related genes and dopamine content levels indicate that Kiss1 may have neuromodulatory effect on dopaminergic neurons. As in mammals, tyrosine hydroxylase encoded by *th1* gene is responsible for dopamine production^23^ and dopamine transporter encoded by *dat* is responsible for reuptake of extracellular dopamine in fish^24^. Therefore, upregulation of *th1* gene expression and dopamine content by Kiss1 administration in the telencephalon-POA could be functionally correlated, while the upregulation of dopamine levels seen in the hypothalamus at 6-h post-administration could be induced by an indirect effect of Kiss1 administration. Further functional characterization of cellular and physiological properties of each dopaminergic neural populations and their association with Kiss1 is necessary to identify the dopaminergic cell population that are selectively sensitive to Kiss1 action. The significant effect of Kiss1 administration on dopamine-related gene expression only appeared at 24-h after administration, while its effect on dopamine content levels was first observed at 30-min post administration. This time gap between gene expression and dopamine content levels suggest that upregulation of dopamine-related genes is due to the secondary effect of Kiss1 on dopamine content levels. We also noticed significant alterations in dopamine levels in all the brain regions examined in the control group. Since all brain specimens were collected at a fixed time (1300 till 1500) of the day, the alteration of dopamine levels is not likely due to the circadian rhythmicity of dopamine release and turnover^25,26^. However, it is possible that the injection itself or the associated handling stress may have affected the dopamine content levels as the dopamine system is known to be influenced by stress^27,28^. On the other hand, in the zebrafish, *th2* has been shown to modulate the synthesis of serotonin (5-HT) in vivo and has tryptophan hydroxylase activity in vitro^29^. In fact, neurons immunoreactive for zebrafish TH2 are highly co-localized with 5-HT in the hypothalamus in the zebrafish^30^. Therefore, downregulation of *th2* expression might have affected 5-HT rather than dopaminergic levels in the telencephalon, optic tectum and hindbrain regions. In addition, because tyrosine hydroxylase is also expressed in noradrenergic neurons^31^, Kiss1 administration may have also stimulated synthesis and release of norepinephrine.

To date, there has been no study that has shown morphological or physiological interaction between habenular Kiss1 and the dopaminergic system in any species. In addition, functional Kiss1Rs are not expressed in brain regions containing dopaminergic neurons in the zebrafish^3^. Although an association between hypothalamic kisspeptin and dopamine has been demonstrated in some species^32–35^, in the present study, administration of the Kiss1 paralogous peptide, Kiss2, which is mainly expressed in the hypothalamus in the zebrafish^9^, had no effect on the expression of dopamine-related genes. Therefore, the effect of Kiss1 on dopamine levels is likely due to the activation of habenular Kiss1-Kiss1R signalling and not Kiss1-Kiss2R signalling. In the brain of zebrafish, *kiss1* and *kissr1* mRNA are expressed in the vHb neurons, although a small amount of *kissr1* mRNA was also detected in microdissected ventral IPN region^9,10^. Kiss1R protein is also expressed along Kiss1 neuronal projections derived from the vHb towards presynaptic terminals at the MR^3,9^. Zebrafish *kissr1* (also referred to as *kiss1rb*) gene has been shown to produce 4 additional alternative splice variants encoding truncated forms of Kiss1R, which are expressed outside of the habenula^3,36^. However, none of the truncated Kiss1R forms have Kiss1 peptides-binding capabilities^36^. Since there is no expression of functional Kiss1R outside of vHb-MR pathway (postsynaptic zone of Kiss1 terminals or interneurons within the MR), Kiss1 may act on Kiss1 neurons in a closed-loop autocrine fashion^10^, which corresponds with the reduced *kiss1* expression in the fish administered with Kiss1. We also found a significant reduction of *npas4a* expression in the vHb following exogenous Kiss1 administration^10^, indicating that administered Kiss1 peptides would have hyperpolarized vHb neurons^13^ through Kiss1R. Hence, the inhibition of habenula Kiss1 neural activities and Kiss1 levels are likely to be induced by negative feedback of exogenous Kiss1 on habenula Kiss1 neurons. However, the exact location of action sites of Kiss1 within Kiss1 neurons (cell coma, axon or presynaptic terminals) and how Kiss1 neural terminal at the MR further transmit their neural activities downstream remain unknown.

To determine the neural activity of dopaminergic neurons, we utilised Npas4, as it has recently been recognized as a sensitive and rapid neuronal activity marker for both excitatory and inhibitory (e.g. glutamatergic and GABAergic) neurons in mammals and zebrafish^21,37–40^. However, we failed to detect co-expression of *npas4a* in dopaminergic neuronal populations following Kiss1 administration. Although Npas4 expression is selectively induced by neuronal activity, it is however, required when activity-dependent synapses are newly formed^21,38^. Therefore, although Kiss1 administration stimulates dopaminergic synthesis, but it may not have induced any synaptic formation in dopaminergic neurons. In addition, in rats, basal levels (without any stimuli) of Fos-related antigen expression is seen only in 10–20% of hypothalamic dopaminergic neurons^33^, and similarly in the zebra finches, basal levels of expression of Fos is seen only in 0.1–2% of preoptic/hypothalamic dopaminergic neurons^41^. Therefore, absence of *npas4a* in dopaminergic neurons could also be due to low levels of expression of neural activity markers in these neurons.

Although dopamine levels in all three macro-dissected brain regions increased following Kiss1 administration, they showed different pattern of responses (time and amplitude) against Kiss1 administration. The increment of *th1* mRNA and dopamine levels in the telencephalic regions at 30-min and 1-h post administration of Kiss1 was the most apparent, while effect of Kiss1 on dopamine levels in the diencephalic region was only seen at 6-h post administration. In mammals, LHb neurons mainly innervate the tail of the ventral tegmental area (VTA, also known as rostromedial tegmental nucleus), which project heavily to midbrain dopamine neurons through the GABAergic pathway^42,43^. In zebrafish, dopaminergic neurons in the periventricular nucleus of posterior tuberculum in the diencephalic cluster are considered a homologue of mammalian midbrain dopaminergic population^44,45^. However, there is neither a direct innervation by Kiss1 fibers nor Kiss1R expression in the diencephalic regions^3,46^. In addition, there was no prominent and rapid effect of Kiss1 on dopamine levels in the diencephalic regions, suggesting that the telencephalic dopaminergic clusters could be the primary action target for habenular Kiss1 neurons. In the present study, our tracer experiment showed putative connections from the MR region to the forebrain as proposed previously^8^. Tracer-labelled nerve fibres were also seen in the mesencephalic and rhombencephalic regions. However, similar distribution of tracer-labelled fibres, except for those in the telencephalon, were also observed when the tracer was implanted into the IPN region, an adjacent region to the MR. Hence, it can be speculated that the fibres labelled in the mesencephalon and rhombencephalon are primarily derived from the IPN due to leakage of the tracer from the MR, but the fibres/tracts labelled in the diencephalon towards the telencephalon are derived from the MR. The tract linking the diencephalon and the telencephalon is known as the medial or lateral forebrain bundle (MFB/LFB), that starts in the telencephalon and extends into two distinct tracts; lateral to the dorsal preglomerular area and medial to the posterior tuberal nucleus in the fish brain^47^. Direct connections between the MR and MFB/LFB have not been shown in the brain of teleosts. However, in rats, the MR-forebrain tract lies in the ventromedial aspect of the MFB and projects to medial forebrain areas^48^. Similarly, in a cartilaginous fish (*Platyrhinoidis triseriata*), retrograde neural tracer injection into the forebrain bundle showed tracer-labelled cells in the superior raphe^49^. These histological analyses indicate possible link between the MR and the telencephalon via the forebrain bundle. Habenular Kiss1 neurons send projections and make putative terminals in proximity of GABAergic and serotonergic neurons in the MR of zebrafish^3,50^. Therefore, it can be postulated that Kiss1 neurons may act on telencephalic dopaminergic clusters via interneurons located in the MR^51^.

Although the physiological role of Kiss1-Kiss1R signalling in habenula neurons remains unclear, expression of Kiss1R (= GPR54) in the habenula has been identified in several fish species and also in mammals^52,53^. In mammals and some non-mammalian species, Kiss1 primarily acts as modulator of reproductive functions via regulation of the hypothalamus-pituitary axis^54^. In mammals, Kiss1 suppresses the activity of the tuberoinfundibular dopaminergic neurons, which has been implicated in prolactin secretion^33,34^. Recently, the potential involvement of Kiss1-Kiss1R signalling in cognitive functions has been reported. In mice, Kiss1 peptide (kisspeptin-13) enhances memory including passive avoidance memory consolidation and also mitigates memory impairment induced by Amyloid-β^55,56^, suggesting possible link between Kiss1 and dopaminergic signalling. However, neither direct association of Kiss1 with nor expression of Kiss1R in midbrain dopaminergic neurons have been reported. Recent accumulated evidences have implicated the role of habenula in spatial and aversive memory processing^57–59^. Further, it is well known that midbrain dopamine system is necessary for associative learning and temporal stability of memories^60,61^. Thus, it can be postulated that Kiss1-Kiss1R signalling could influence cognitive functions via indirect action through the habenula-VTA pathway in mammals.

**Figure 8:**
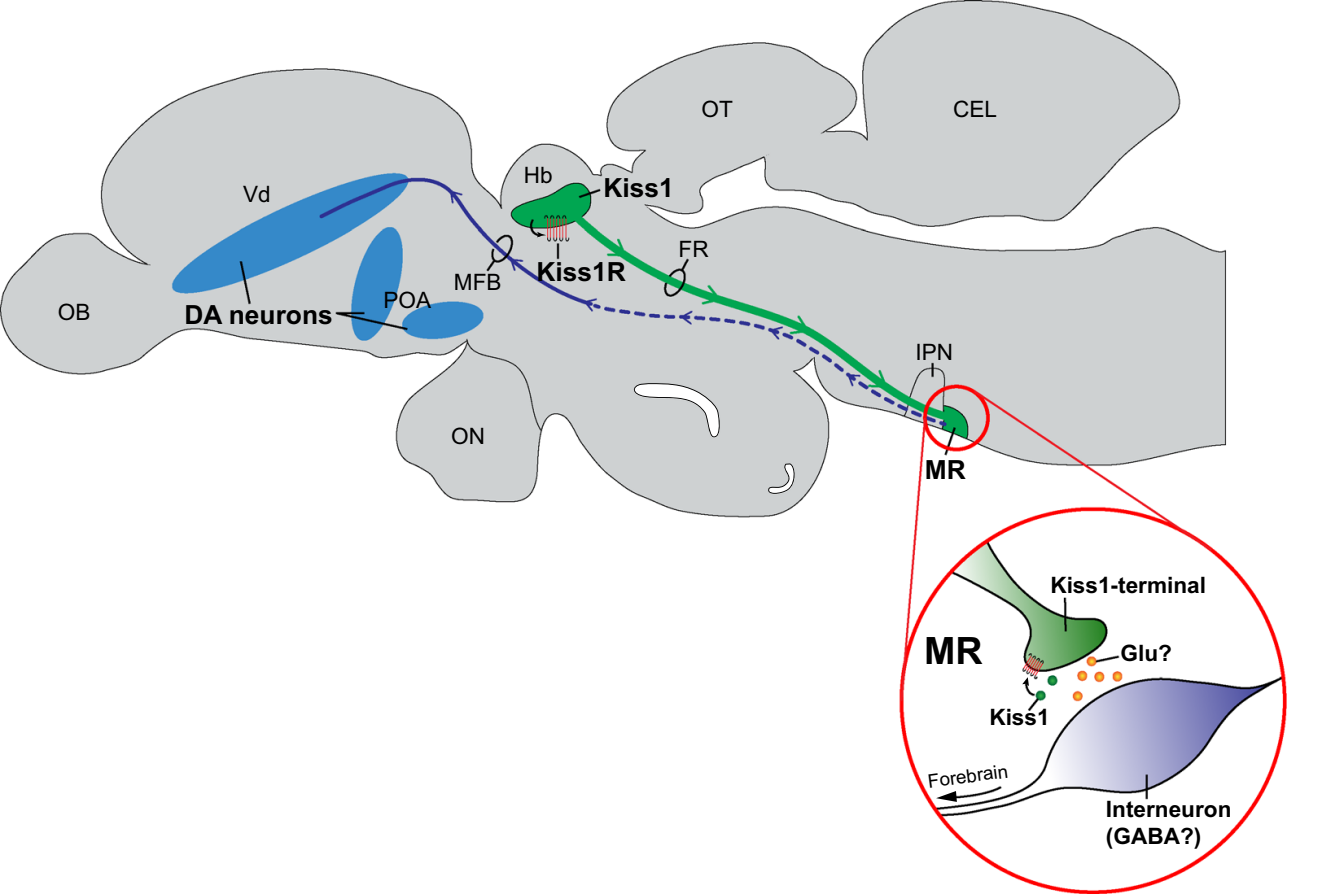
Schematic drawing illustrating putative connection from the habenula Kiss1 towards telencephalic dopaminergic neurons in the brain of zebrafish. Habenula Kiss1 neurons send projection and terminate at the median raphe (MR) through the fasciculus retroflexus (FR). Habenula Kiss1 neuronal activity is controlled by autocrine feedback of Kiss1 via co-expressing Kiss1R. At the MR, inhibition of Kiss1 neurons may influence on postsynaptic glutamatergic (Glu) transmission on interneurons such as GABAergic neurons (in red circle). Neurons receive Kiss1 inputs within the MR further send projections towards the telencephalon via the medial forebrain bundle (MFB) and possibly act on the telencephalic dopaminergic (DA) cells. *OB* olfactory bulb, *Vd* ventral telencephalon, *POA* preoptic area, *ON* optic nerve, *Hb* habenula, *OT* optic tectum, *CEL* cerebellum, *IPN* interpeduncular nucleus.

In summary, the present study demonstrates stimulatory effect of centrally administered Kiss1 peptides on dopamine levels in the telencephalic regions. In fish administered with Kiss1, neural activity and *kiss1* gene expression were significantly decreased, suggesting autocrine and negative feedback regulation of Kiss1 neurons. Since the habenular Kiss1 neurons form terminals in the MR^11,46^ and our tracer study showed potential link between the MR and the telencephalon via the MFB, we speculate that habenular Kiss1 negatively regulates forebrain dopamine neurons through interneurons in the MR (Fig. 8).

## Materials and methods

### Animals

Sexually mature (> 6 months old) male, AB wild-type zebrafish (*Danio rerio*) were obtained from the zebrafish facility at the Institute of Molecular and Cell Biology, Singapore. Transgenic, Tg(*dat*:EGFP) zebrafish line, in which the enhanced green fluorescent protein (EGFP) is expressed in dopaminergic neurons under the control of cis-regulatory elements of the dopamine transporter (*dat*) gene^62^ was generously provided by Professor Marc Ekker from University Ottawa, Canada. Tg(*kiss1*:mCherry) zebrafish line, in which the mCherry is expressed in habenular Kiss1 neurons under the control of *kiss1* gene promoter^46^, was generously provided by Professor Wei Hu, Institute of Hydrobiology, Chinese Academy of Sciences, Wuhan, China. Fish were maintained in freshwater aquaria at 26 ± 0.5 °C with controlled natural photo regimen (14/10 h; light/dark) at the BRIMS (Brain Research Institute) fish facility, Monash University Malaysia. Fish were fed Tetramine flaks (Tetra, Germany) twice daily. All fish were acclimatized for two weeks before experiments.

### Ethics statement

This study was carried out in strict accordance with the recommendations in the Guidelines to promote the wellbeing of animals used for scientific purposes: The assessment and alleviation of pain and distress in research animals (2008) by the National Health and Medical Research Council of Australia (https://www.nhmrc.gov.au/guidelines-publications/ea18). Fish were anesthetised by using either 0.01% benzocaine exposure or rapid-chilling, which have been acceptable forms of anaesthesia and euthanasia in zebrafish^63^. All experimental protocols were approved by the Animal Ethics Committee of Monash University (Approval nos. MARP/2013/095 and MARP/2017/022).

### Intracranial administration of Kiss1 peptides and sample collection

Central administration of Kiss1 peptides was performed as described previously in Kizil and Brand (2011) and Ogawa et al*.* (2012)^10,64^. For whole brain gene expression assay, AB fish were anesthetized by immersion in 0.01% benzocaine (Sigma, USA) solution and intracranially administered with 1 μL of either kisspeptin1-15 (Kiss1; pyroglut-NVAYYNLNSFGLRY-NH_2_; Open Biosystems) or kisspeptin2-10 (Kiss2; FNYNPFGLRF-NH_2_; BioGenes) with two different doses (10^–12^ and 10^–9^ mol/fish in distilled water). For injection, the cranial bone close to the habenula above the left side of the anterior part of the optic tectum was incised using a sterilized barbed-end needle (BD Precision Glide 30G × 0.5″, BD Medical, New Jersey) (Fig. 1A), and through this incision,

Kiss1 solution was administered using a heat-pulled glass capillary micropipette attached with a microinjector (IM-9B, Narishige, Tokyo, Japan) (Fig. 1B). For control, the same volume of distilled water was administered. Fish in sham group received the puncture procedure without any administration.

### Real-time PCR for dopamine-related genes and *kiss1* gene

Expression of dopamine-related genes: *th1*, *th2* and *dat*, and *kiss1* mRNA levels were measured in whole and macro-dissected brains using real-time PCR. For whole brain, following intracranial injection, fish were euthanized by rapid-cooling in ice cold water 30-min, 1-h, 3-h, 6-h or 24-h after administration (n = 7/group), while for the macro-dissected samples, the brains were dissected 24-h after administration (n = 10/group). For the fish treated with sham and Kiss2, the brains were collected 24-h after administration. All sample collections were conducted consistently between 13:00–14:00 for every experimental protocol. For regional gene expression assay, the whole brain was further macro-dissected into five different brain regions: telencephalic region, preoptic-pretectum region, optic tectum, midbrain-hypothalamic region, rhombencephalic region (Fig. 2A) under a stereoscopic microscope. Whole or macro-dissected brain samples were homogenised in TRIzol reagent (Life Technologies), and total RNA were isolated and dissolved in 10 μL of RNase-free water. The total RNA was reverse transcribed to cDNA using High Capacity cDNA Reverse Transcriptase kit (Applied Biosystems) in a 20 μL reaction mixture according to the manufacturer’s instruction. Real-time PCR were performed using the Applied Biosystems StepOne (PE Applied Biosystems, CA, USA) in a total volume of 10 μl reactions contained 1 × POWER SYBR Green PCR Master Mix (PE Applied Biosystems, CA, USA), 0.2 μM each gene-specific forward and reverse primers (Table 1) and 1 µl cDNA. The reaction was programmed for 50 °C for 2-min, 95 °C for 10-min and 40 cycles of 95 °C for 15-s and 60 °C for 1-min, followed by a dissociation stage. The threshold cycle (Ct) of each gene of interest was obtained and normalised against mRNA expression levels of eukaryotic translation elongation factor 1 alpha 1, like 1 (*eef1a1l1*). The data were then analysed according to 2^-ΔΔCt^ relative gene expression quantification.

**Table 1:** Sequence of primers used for real-time PCR.

Gene	GenBank accession no	Forward (5′–3′)	Reverse (5′–3′)
*th1*	BC163630.1	GCACCTGTCGGATGTTAGCA	ATTTGACCTCCTTTGTGGTTTTG
*th2*	NM_001001829.1	AAGAGCAAGAGAAGCAGTGGAGA	TGAAGATGTCGGTGTCGGAG
*dat (slc6a3)*	NM_131755.1	CATTTACCCAGAAGCCATTGC	GCGTGTCGATTCCTAAAGTCA
*kiss1*	AB245404	ACAAGCTCCATACCTGCAAGTG	AATACTGAAAATGCCCAGAGGG
*eef1a1l1*	NM_131263.1	ACCCTCCTCTTGGTCGCTTT	CCGATTTTCTTCTCAACGCTCTT

### Dopamine quantification

Dopamine levels in macro-dissected brain samples were determined using liquid chromatogram (LC)-double mass spectrometry (MS/MS) (LC–MS/MS) following the procedures previously developed by Kim et al. (2016)^65^ with some modifications. AB fish were first administered with Kiss1 at the doses of 10^–12^ and 10^–9^ mol/fish or saline (n = 6 /group), and the brains were collected at 30-min, 1-h, 3-h, and 6-h after Kiss1 administration. The dissected brain was further macro-dissected into three different brain regions (Fig. 3A) based on the classification of dopaminergic cell clusters demonstrated by Paula et al. (2010)^44^ under a stereoscopic microscope. Telencephalic complex consists of the olfactory bulb, subpallium, and anterior preoptic area; Preoptic-pretectal complex consists of those in the preoptic area, pretectum, and ventral thalamus; Diencephalic complex consisted of the hypothalamic area, caudal hypothalamic region and the midbrain. Details of chemicals used, sample preparation procedures and condition of LC–MS/MS are described below:

#### Chemicals

All solvents were of high-performance LC grade. Acetonitrile, formic acid and dopamine were obtained from Sigma Aldrich (St. Louis, MO, USA). Working solutions were prepared in ultrapure water provided by a Milli-Q system (Millipore, Bedford, MA, USA). Each analytical stock solution (1 mg/ mL) was prepared in 0.111 M ascorbic acid in a 1:1 mixture solution to prevent oxidation and stored at 80 °C. A working internal standard solution (buffer A), 20 pg/μL of isoproterenol (Nacalai, Japan) was prepared in buffer B (50% acetonitrile,0.1% formic acid, and 0.111 M ascorbic acid) immediately before analysis. Stock of 10 mg/mL dopamine standard were diluted in buffer A, followed by calibration curve in which dopamine stock was diluted in buffer A according to corresponding concentration (0.025, 0.25, 1.25, 1.5, 2.0, 2.5, 4.0 pg/μL).

#### Sample preparation

The macro-dissected brain was homogenized in 80 µL of buffer B supplemented with 20 pg/µL of the internal standard solution using micropestle for 60s. For the investigation of selectivity, matrix effects and sensitivity, authentic homogenized filtered samples were used. The sample preparation was processed briefly in an ice bath to prevent the possible degradation of the analytes. Five microliter of the samples were injected into the LC–MS/MS system. Quality control was done by protein quantification against bovine serum albumin using BioRad Protein Assay dye (Cat#: 5000006, BioRad) according to the manufacturer’s instructions. Dopamine levels in the brain tissue homogenate were normalized by total protein content levels. The assay was read on Labsystems multiskan 352 plate reader (Thermo Fisher Scientific) at a wavelength of 595 nm.

#### LC analysis

LC–MS analysis was conducted using a 1240 infinity LC system and 6460 triple quadrupole MS/MS (Agilent Technologies, Santa Clara, CA, USA) coupled with a 1240 infinity extra binary pump and degasser (Agilent Technologies) used for operating an additional column. Data were processed using the MassHunter software (B. 04.00, Agilent Technologies). The LC–MS analysis setting was determined based on Kim et al. (2016)^65^. The mobile phase consisted of 0.1% formic acid in 5 mM ammonium formate and 0.1% formic acid in acetonitrile for the entire analysis and the flow rate for both the loading pump and the analytical pump was 300 mL/min. The temperature of the auto- sampler was set to 4 °C. The sample was loaded onto separation column SB C18 (2.1 mm—250 mm, 3 mm Agilent) on valve position 1. Gradient conditions 0–1.0 min, 5% B; 1.0–1.8 min, 5–90% B; 3.5 min, 90. Column was maintained at 45 °C.

#### MS/MS condition

The MS/MS system was operated using polarity-switching electrospray ionization (ESI). The optimal conditions for the analysis were determined based on Kim et al. (2016)^65^ as follows: capillary voltage, 4.5 kV; nebulization pressure, 35 psi; temperature of drying gas, 350 °C; drying gas flow, 10 L/min; sheath gas temperature, 250 °C; sheath gas flow, 5 L/min; and nozzle voltage, 0.5 kV. The time segment with the start time of 2.5 min was applied to prevent flowing interference into the mass spectrometer. The electron multiplier voltage for the run time of 2.5–4.4 min was set to 40 V in the negative ESI. Multiple reaction monitoring transitions were chosen for dopamine and internal standards.

### In situ hybridization of *npas4a*

Effect of Kiss1 treatment on neural activities in the habenula and dopaminergic neurons were examined by expression of neural activity marker, *npas4a* expression, which were examined in the brain of AB-wild type or Tg(*dat*:EGFP) fish, respectively. The whole brain (n = 6 fish/group) was dissected 30-min after the Kiss1 administration and fixed in buffered 4% paraformaldehyde for overnight at 4 °C and then expression of *npas4a* were examined by in situ hybridization. For *npas4a* mRNA expression in the habenula, coronal sections (10 µm) (n = 6 for control and Kiss-treated) of wild-type AB male were hybridized with DIG-labelled *npas4a* riboprobes (737nt, GenBank accession number: NM_001045321) for 16 h at 55 °C. DIG-labelled *napas4a* mRNA was detected with either anti-DIG-AP or anti-DIG-POD antibody (Roche) followed by chromogenic development with NBT/BCIP or amplification using TSA Plus Cyanine 3 System (Perkin Elmer/AKOYA Biosciences), respectively. After TSA amplification of *npas4a* mRNA signals, EGFP signals were further enhanced with a rabbit anti-GFP antibody (1:500 dilution, Millipore Cat# AB3080, RRID:AB_91337) followed by incubation with Alexa Fluor 488-labeled anti-rabbit IgG (1:500 dilution, Thermo Fisher Scientific).

### Neural tracer application into the median raphe

To examine the possible target of the Kiss1-terminus region in the median raphe (MR), a lipophilic tracer, NeuroVue Jade dye (green emitting dye; excitation maximum, 478 nm; emission maximum, 508 nm)-coated filter (Polysciences, Inc., Warrington, PA) was applied to the MR region in the brain of Tg(*kiss1*:mCherry) fish (n = 3). A nylon filter coated with dye was cut in a small pieces in triangular thin-strips (approx. 0.1 mm width × 1 m length) using an agitate scissors under a stereoscopic microscope, which allows to apply the tracer into the target brain region more precisely with less leakage of tracer from the target area^66^. As negative controls for tracer applications, the brain without tracer implantation (n = 1) and the brain with tracer application into the IPN region (n = 3) were prepared.

The brain of Tg(*kiss1*:mCherry) fish was dissected and fixed in buffered 4% paraformaldehyde for overnight at 4 °C. The whole brain was cut into half longitudinally using a surgical knife and a dye-coated thin-strip was inserted to the mCherry-labelled MR region via sharp forceps under a florescence stereoscopic microscope (Nikon, SMZ1500). After dye application, the brain specimen were placed in buffered 4% paraformaldehyde and incubated at 37 °C for 3-days. For observation, the brain specimen was cryoprotected in 20% sucrose and embedded in Tissue Tek OCT compound (Sakura Finetechnical, Tokyo, Japan). The sagittal brain sections (15 μm thickness) were cut using a cryostat and were thaw-mounted onto 3-aminopropylsilane-coated glass slides.

### Microscopy

Acquisition of the microscopy images were carried out as described previously^67^. Images for DIG-labelled *npas4a*-expressing cells were captured with a Zeiss MIRAX slide scanning system (Carl Zeiss, Germany) using the Mirax Viewer Image Software (3DTech, Budapest, Hungary). Fluorescent images were captured separately with a fluorescence microscope (ECLIPS 90i; Nikon, Tokyo, Japan) that was attached to a digital cooled CCD camera (DMX-1200C, Nikon) with appropriate excitation filters for Cyanine 3, Alexa Fluor 488, NeuroVue Jade and mCherry, and computer software (NIS Elements D3.2; Nikon) was used to superimpose the two images. The red channel was then converted to magenta, and brightness and contrast adjustments were made in Adobe Photoshop CC (Adobe, San Jose, CA, USA).

### Data and statistical analysis

All data are presented as means ± SEM, and statistical analyses were performed using independent t-test to observe any statistical significance between controls and experimental groups. Statistical differences among experimental groups were analyzed using one-way or two-way ANOVA, followed by a Tukey’s multiple comparison or a Dunnett’s multiple comparison *post-hoc* test (Prism 8, GraphPad Software, CA). Differences were considered statistically significant at *P* < 0.05. Sample size were calculated using G*Power 3.1 (https://www.psycho.uni-duesseldorf.de/abteilungen/aap/gpower3/)^68,69^. Animals were randomly drawn from the tank immediately before testing, and the order with which phenotypes were tested was randomized via generation of random numbers using the randomization tool in https://www.randomization.com/. No exclusion criteria were pre-determined.
